# Bioelectric Dysregulation in Cancer Initiation, Promotion, and Progression

**DOI:** 10.3389/fonc.2022.846917

**Published:** 2022-03-14

**Authors:** Maulee Sheth, Leyla Esfandiari

**Affiliations:** ^1^ Department of Biomedical Engineering, University of Cincinnati, Cincinnati, OH, United States; ^2^ Department of Electrical Engineering and Computer Science, University of Cincinnati, Cincinnati, OH, United States; ^3^ Department of Environmental and Public Health Sciences, University of Cincinnati, Cincinnati, OH, United States

**Keywords:** bioelectricity, extracellular vesicles, tumor microenvironment, membrane potential, carcinogenesis, piezo channels

## Abstract

Cancer is primarily a disease of dysregulation – both at the genetic level and at the tissue organization level. One way that tissue organization is dysregulated is by changes in the bioelectric regulation of cell signaling pathways. At the basis of bioelectricity lies the cellular membrane potential or V_mem_, an intrinsic property associated with any cell. The bioelectric state of cancer cells is different from that of healthy cells, causing a disruption in the cellular signaling pathways. This disruption or dysregulation affects all three processes of carcinogenesis – initiation, promotion, and progression. Another mechanism that facilitates the homeostasis of cell signaling pathways is the production of extracellular vesicles (EVs) by cells. EVs also play a role in carcinogenesis by mediating cellular communication within the tumor microenvironment (TME). Furthermore, the production and release of EVs is altered in cancer. To this end, the change in cell electrical state and in EV production are responsible for the bioelectric dysregulation which occurs during cancer. This paper reviews the bioelectric dysregulation associated with carcinogenesis, including the TME and metastasis. We also look at the major ion channels associated with cancer and current technologies and tools used to detect and manipulate bioelectric properties of cells.

## Introduction

Carcinogenesis, also termed oncogenesis or tumorigenesis, is rooted in two major theories or hypotheses, both significantly different from one another. The somatic mutation theory (SMT), which has been prevailing in cancer research for more than sixty years proposes that the origin of cancer can be explained by an accumulation of several DNA mutations in a single somatic cell. Tumor development is then a multistep process where successive mutations produce advantageous biological compatibilities (1). The SMT explains many features of cancer such as hereditary cancers and the success of gene-targeting cancer therapies (2). However, non-genotoxic carcinogens which induce cancer without any DNA modifications (3) and the absence of mutations in some tumors (4) contradict this theory. Alternatively, the tissue organization field theory (TOFT) proposed in 1999, hypothesizes that carcinogenesis is a problem of tissue organization instead of having a cellular level origin. Here, the carcinogenic agents disrupt the reciprocal interactions between cells that maintain tissue organization, repair, and homeostasis, hence creating altered microenvironments which allow the parenchymal cells to exercise their ability to proliferate and migrate (5).

Bioelectric regulation is an important mechanism of cell communication and dysregulation of this mechanism can result into alterations in tissue organization, fitting the tissue organization field theory of carcinogenesis. While bioelectricity has been extensively studied in cells with neural origins, its role in non-neural cell activity and functionality has only emerged more recently. With advances in understanding the underlying bioelectric mechanisms of cancer and development of molecular tools to measure and control these electric fields, we are now able to better identify the role of bioelectric signaling in carcinogenesis. Another important mechanism that facilitates intercellular communication for the maintenance of tissue homeostasis is the production and release of extracellular vesicles (EVs) by cells of different tissue types. Cancer-derived EVs play a role in all steps of carcinogenesis by mediating the communication between cancer cells and non-cancer cells as well as malignant cells and non-malignant cells within the tumor microenvironment (TME) (6). Furthermore, the production of EVs is aberrant during cancer which in turn plays an important role in disturbing the bioelectrical signaling pathways between cells.

Several review papers (7–10) focusing on the bioelectric control of one or the other aspect of cancer, such as migration or metastasis, have been published. In this paper, we provide a more extensive review of bioelectric regulation in multiple cancer processes including initiation, promotion, the tumor microenvironment, and metastasis. We also look at the major ion channels implicated in cancer and current technologies and tools used to measure and manipulate bioelectric properties of cells *in vivo*.

## Bioelectricity and Endogenous Electric Fields – An Overview

Membrane potential (V_mem_) is an electrical property associated with any cell, specific to its origin and function. The electric nature of the membrane potential produces endogenous electric fields (EFs) due to the segregation of charges by molecular machines such as pumps, transporters and ion channels that are primarily located in the plasma membrane of the cell (11). These transmembrane voltage gradients have been established to control not only neural signaling *via* gap junctions, but also cell proliferation, migration, differentiation, and orientation in both, excitable and non-excitable cells (12, 13).

**Figure 1:**
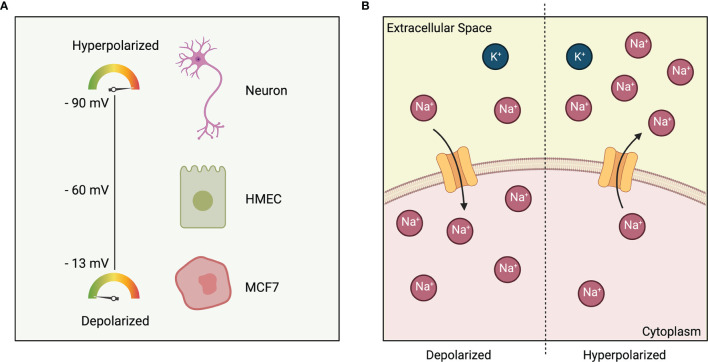
**(A)** Polarization of cells based on cell type. Excitable cell such as neurons have a membrane potential of -90 mV. Non-excitable cells such as HMEC: Human Mammary Epithelial Cell and MCF7: Estrogen-receptor-positive breast cancer cell line are at -60 mV and -13 mV respectively. **(B)** Depolarized cell state (left) indicated by a more positive charge in the cytoplasm relative to the extracellular space. Hyperpolarized cell state (right) indicated by a less positive charge in the cytoplasm relative to the extracellular space.

Depending on the presence of relative charges, all excitable and non-excitable cells possess an electric gradient across their plasma membrane (**Figure 1A**). When the cytoplasm becomes more positively charged relative to the extracellular space, the cell is said to be depolarized and will have a less negative V_mem_. When the cytoplasm becomes more negatively charged relative to the extracellular space, the cell in said to be hyperpolarized and will have a more negative V_mem_ (**Figure 1B**). It is worthwhile to note that V_mem_ is not only a key intrinsic cellular property, but also an integral part of the microenvironment where it acts both, spatially and temporally, to guide cellular behavior (9). It does so by enabling the cells to make decisions based on the states of their neighbors (14). Physiological V_mem_ can range from -90 to -10 mV, depending on the cell type and physiological state (13, 15). Furthermore, as V_mem_ is primarily established by ion channels that are gated post-translationally, two cells that are in the exact same genetic and transcriptional states could theoretically be in very different bioelectric states (16).

## Bioelectricity in Cancer Processes

Bioelectric properties of cells and the electrical state of cells in the microenvironment are known to control several key behaviors of relevance to cancer (17–24). Here we first introduce some major ion channels implicated in cancer. Then we look at the role of bioelectricity in cancer initiation and progression, the tumor microenvironment, and migration and metastasis.

### Ion Channels and Cancer

Ion channels are membrane proteins that create ionic concentration gradients by regulating the flow of ions across the plasma membrane. The primary function of ion channels is to maintain cellular homeostasis by regulating the inward and outward ion flux, but they are also higher order regulators of many downstream molecular signaling pathways (7). The four main ions that play a role in establishing the resting V_mem_ of a cell are: Ca^2+^, Na^+^, K^+^, and Cl^-^. The Goldman equation links the overall transmembrane potential to the concentrations and permeabilities of various ion species. The resting potential V_mem_ depends on the internal and external K^+^, Na^+^, and Cl^-^ concentrations, ambient temperature, and permeability of each ion specie. Alterations in ion channel expression and activity are associated with the initiation, proliferation, and metastasis of cancer cells (21, 25). For instance, there is a host of ion channels whose expression is dysregulated in cancer cells and have been found to be associated with a metastatic phenotype (7). Here, we summarize major ion channels responsible for the disruption of homeostasis and aberrant activation of downstream signaling pathways in cancer including voltage-gated cation channels (Ca_V_, Na_V_, K_V_), mechanosensitive cation channels, transient receptor potential (TRP) channels, and chloride channels (CLCs). Several review papers focusing extensively on ion channels implicated in cancer can be found in literature (26–29). It is worthwhile to note that disruption in expression of these ion channels leads to deregulation in a host of different signaling pathways in cancer (27–30). Prominent ones include the mitogen-activated protein kinase (MAPK) pathways, ERK and JNK signaling pathways, Wnt/ß-catenin pathway, PI3K/Akt pathway, Notch signaling, and the Rac and Rho pathways.

#### Calcium Channels

Voltage-gated calcium channels (VGCCs) and transient receptor potential (TRP) ion channels are primary channels facilitating Ca^2+^ ion diffusion. VGCCs are present in human breast cancer cells but not in normal human mammary epithelial cells (HMECs) (31). Berzingi et al. studied the effect of calcium ions on cell proliferation. Upon 5 days of culture, it was found that MCF7 breast cancer cells showed almost no growth in a culture medium without Ca^2+^ ions compared with cells growing to nearly 100% confluence in a medium containing 2 mM Ca^2+^ ions. Furthermore, blocking external Ca^2+^ ions from entering the cell through voltage-gated calcium channels using Verapamil indicated that cell growth was substantially inhibited in MDA-MB-231, breast cancer cells (32). The intracellular calcium concentration is also integral for cancer cell metastasis since it regulates the cell cytoskeletal dynamics, protease activity, cell volume, and pH – all of which play a role in migration and invasion of cancer cells (33–36). Calcium is also involved in driving ECM degradation and cell invasion by promoting epithelial-mesenchymal transition (EMT) pathways and the activity of matrix metalloproteinases (37, 38). Furthermore, multiple TRP channels are regulated differently in various cancers. Expression levels of TRPC3 in some breast and ovarian tumors (39) and TRPC6 in breast, liver, stomach cancers and in glioma are elevated (40). In non-small-cell lung carcinoma cells, Ca^2+^ entry mediated by TRPC1 and its associated signaling was found to activate the Pl3K/Akt and MAPK downstream pathways and simulate proliferation (41). Some TRP channels including TRPC1 (42), TRPC3 (43), TRPC6 (44–46), TRPM2 (47–49), and TRPM8 (50, 51) also simulate apoptosis by increasing Ca^2+^ activity. Consequent increase in TRPC6-mediated Ca^2+^ entry has also been found to alter the Notch pathway, leading to tumorigenesis in human glioblastoma multiforme (GBM) and GBM-derived cell lines (52). TRPV4 is also a critical regulator of the Rho signaling pathway involved in cancer cell adhesion and migration (53).

#### Sodium Channels

Cancer cells can effectively use Na^+^ flux to indirectly promote a metastatic phenotype. For instance, changes in Na^+^ flux can create localized areas of depolarization that can drive the movement of Ca^2+^ and H^+^ ions. Activity of Na^+^/Ca^2+^ exchangers located in the plasma membrane of cells has also been linked to favor ECM degradation and cell invasion, as has been demonstrated in MDA-MB-231 breast cancer cells that overexpress a voltage gated sodium channel (VGSC) (54). The expression of Na_V_1.7 also promotes cellular invasion at the transcriptional level by epidermal growth factor (EGF) and EGF receptor (EGFR) signaling *via* the ERK1/2 pathway (55). In colon cancer cells, Na_V_1.5 activity and the subsequent depolarization have been found to play a role in the induction of invasion-related genes through the MEK, ERK1/2 pathway (56, 57). Furthermore, a sodium-channel SCN5A has been identified as a key regulator of a genetic network that controls colon cancer invasion (57). The activity of some sodium channels has also been shown to further simulate the expression of more sodium channels in prostate and breast cancer cell lines. This allows the cells to substantially increase ion flux by creating a positive feedback loop of channel activity-induced channel expression (58). Finally, changes in the intracellular Na^+^ concentration can also alter cellular pH (10). A decrease in the pH surrounding a tumor is known to influence cell adhesion *via* the formation of integrin-mediated focal adhesion contacts (59–61).

#### Potassium Channels

K^+^ ions predominantly move from the intracellular to extracellular space through their channels to maintain the steady state resting potential of a cell. K^+^ indirectly affects the V_mem_ by driving the entry of Ca^2+^ into the cell. At the same time, the proliferation of some tumor cells is dependent on voltage-gated potassium channels (62–67) that alter cell volume by affecting K^+^ flow. A variety of tumor cells express K_V_10.1 (68, 69) or K_V_11.1 (HERG) (70) or both channels. The K^+^ channel EAG has been found to be expressed in 100% of cervical cancer biopsies analyzed and overexpression of EAG in human cells has been shown to increase cell proliferation in culture (71, 72). Furthermore, overexpressing K^+^ channels in breast cancer cells has been found to drive cell migration mediated by cadherin-11 and MAPK signaling (73). Calcium-dependent K^+^ channel K_Ca_3.1 also promoted proliferation by directly interacting with ERK1/2 and JNK signaling pathways (74). Finally, Ca^2+^ flow through TRPM8 regulates activity of Ca^2+^-sensitive K^+^ channels such as K_Ca_1.1, which plays a role in migration (75, 76). In breast cancer cells, overexpression of TRPM8 increased the metastatic potential *via* activation of the AKT glycogen synthase kinase-3 ß (GSK-3ß) pathway (77).

#### Chloride Channels

Chloride is the main anion that accompanies the transport of cations such as calcium, sodium, and potassium. Chloride channels play an important role in cancer cell migration due to their role in maintaining cell volume (78). Cl^-^ channels have been revealed to have a role in glioblastomas from studies in glioma cell lines (79, 80). Studies of human prostate cancer cell lines have also shown chloride channels to play a role as key regulators of proliferation through cell size regulation (81). Chloride ion channel-4 Cl^-^/H^+^ exchanger has been found to enhance migration, invasion, and metastasis of glioma and colon cancer cells by regulating the cell volume (65). For instance, genetic knockdown of ClC-3 has been found to substantially reduce migration in glioma cells (82).

#### Piezo Channels

Piezo channels are non-selective Ca^2+^-permeable channels whose gating can be simulated by several mechanical stimuli affecting the plasma membrane, including compression, stretching, poking, shear stress, membrane tension, and suction (83–85). A recent study has also demonstrated that Piezo channels show significant sensitivity to voltage cues and thus can also be viewed as important members of the voltage-gated ion channel family (86). Two major piezo channels – Piezo1 and Piezo2 have been identified which are mainly expressed in different tissues. Piezo channels are overexpressed in several cancers, such as breast, gastric, and bladder, whereas in other cancers, their downregulation has been described. Several studies conducted *in vitro* and *in vivo* have demonstrated that the activation of Piezo channels can drive a Ca^2+^ influx, thus modulating key Ca^2+^-dependent signaling pathways associated with cancer cell migration, proliferation, and angiogenesis (87). Overexpression of Piezo1 has also been found to promote prostate cancer development through the activation of the Akt/mTOR pathway (88). Furthermore, the mechanistic effects of Piezo2 are associated with a Ca^2+^-dependent upregulation of Wnt11 expression which enhances the angiogenic potential of endothelial cells in cancer *via* ß-catenin-dependent signaling (89).

### Cancer Initiation and Promotion

Resting potential established by ion channel and pump proteins is important for determination of differentiation state and proliferation. One way that carcinogenesis occurs is due to the disruption of electrical gradients, or the mechanisms by which they are perceived by cells (24). V_mem_ is an important non-genetic biophysical aspect of the microenvironment that regulates the balance between normally patterned growth and carcinogenesis (7). Cancerous and proliferative tissues are generally more positively charged or depolarized than non-proliferative cells (90, 91). V_mem_ values from -10 to -30 mV correspond to more undifferentiated, proliferative, and stem-like cells (92). For instance, the resting membrane potential in normal human mammary epithelial cells (HMEC) is -60 mV. This value goes up to -13 mV in breast cancer cells isolated from patients (93). Berzingi et al. experimentally compared V_mem_ in HMEC and two different invasive ductal human carcinoma cell lines, MCF7 (estrogen-receptor-positive) and MDA-MB-231 (estrogen-receptor-negative). The results indicated that MCF7 and MDA-MB-231 cells are 30.4 mV and 27.3 mV more depolarized in comparison to HMEC cells, respectively. It was also seen that HMEC grew at a much slower rate compared to MCF7 and MDA-MB-231 (32).

Lobikin et al. used a Xenopus tadpole model to confirm the role of ion flow in oncogenesis *in vivo* by investigating the consequences of depolarizing select cell groups (67). Embryos were exposed to glycine-gated chloride channel (GlyCl) activator ivermectin to control the membrane potential of a widely distributed, sparse population of cells expressing the GlyCl channel. The membrane potential of these specific cells could be set to any desired level by manipulating external chloride levels following ivermectin treatment. Tadpoles whose cells were depolarized were seen to exhibit excess melanocytes with a much more arborized appearance and colonize areas normally devoid of melanocytes, such as around the eyes and mouth. It was also shown that depolarization induces the up regulation of cancer relevant genes such as Sox10 and SLUG (94). Furthermore, susceptibility to oncogene-induced tumorigenesis was shown to be significantly reduced by forced prior expression of hyperpolarizing ion channels indicating that bioelectric signaling of the cellular microenvironment can both, induce and suppress, cancer-like cell behavior.

V_mem_ has been suggested as a cancer biomarker due to its role as an early indicator of tumorigenesis and is associated with tumors of diverse molecular origin (95–98). Induced tumor like structures (ITLS) can be formed in Xenopus and zebrafish embryos by mis-expressing mammalian oncogenes (Gli1, Xrel3 and KRASG12D) and mutant tumor suppressors (p53Trp248). ITLS’s are formed as a result of genetic interference with signaling pathways altered in several types of cancer including basal cell carcinoma, lung cancer, leukemia, melanoma, and rhabdomyosarcoma (99–102). Fluorescence reporters of V_mem_ in the injected animals have been found to reveal unique depolarization of tumors and increased sodium content compared to healthy tissues (7, 103). Moreover, depolarized foci have a higher success rate in predicting tumor formation as compared to cancer specific antigen level in the serum. For instance, Chernet and Levin found that depolarized foci, while present in only 19-30% of oncogene-injected embryos, predict tumor formation with 50-56% success rate as compared to prostate specific antigen level in the serum, which when used as a biomarker for prostate cancer, has a 29% predictive value (104, 105).

Recently, Carvalho developed a computational model of cancer initiation, including the propagation of a cell depolarization wave in the tissue under consideration (106). This model looks at an electrically connected single layer tissue in two and three dimensions and simulates ion exchange between cells as well as between cells and the extracellular environment. It was seen that a polarized tissue with cells in quiescent state tends to change state if a large enough perturbation changes its homeostatic conditions, such as a carcinogenic event. The induced depolarized state is able to then propagate to neighboring cells in a wave like manner. The developed model shows the importance of community effects associated with cell electrical communication leading to both, short- and long-range influences and ultimately, cancer.

### The Tumor Microenvironment

The microenvironment functions to guide the cell through space and to direct tissue growth through time. It also plays a significant role in the physiological outcome of a given V_mem_ input. The tumor microenvironment (TME) is a complex entity and consists of multiple cell types embedded in the extracellular matrix (ECM), including immune cells, endothelial cells and cancer associated fibroblasts (CAFs) which communicate with cancer cells and with other CAFs during tumor progression (107). One way this communication is mediated is by a plethora of bioactive molecules, including proteins, lipids, coding and non-coding RNAs, and metabolites, which are secreted into extracellular vesicles (EVs) (108, 109).

The mechanical microenvironment impacts bioelectric regulation and cell proliferation (9). An early indication of this were studies in the late 1900s which found that cells within a low cell density (fewer cell-cell contacts) exhibited reduced proliferation (110) and that cells in a confluent monolayer are more hyperpolarized than individual cells (111, 112). Similarly, chemical components of the cellular microenvironment have the ability to impact cell phenotype. Factors such as hypoxia (113) and pH (114) have been demonstrated to drive cancer progression. Moreover, hypoxic tumors exhibit more aggressive phenotypes. Tumor cells under hypoxia can produce a secretion partly in the form of EVs that modulates the microenvironment to facilitate tumor angiogenesis and metastasis (115). V_mem_ thus functions at the interface of chemical and mechanical signals by creating an electrical gradient across cells, which in turn gates voltage-sensitive channels. This creates a tightly connected communication pathway between a cell and its microenvironment (9, 116–119).

**Figure 2:**
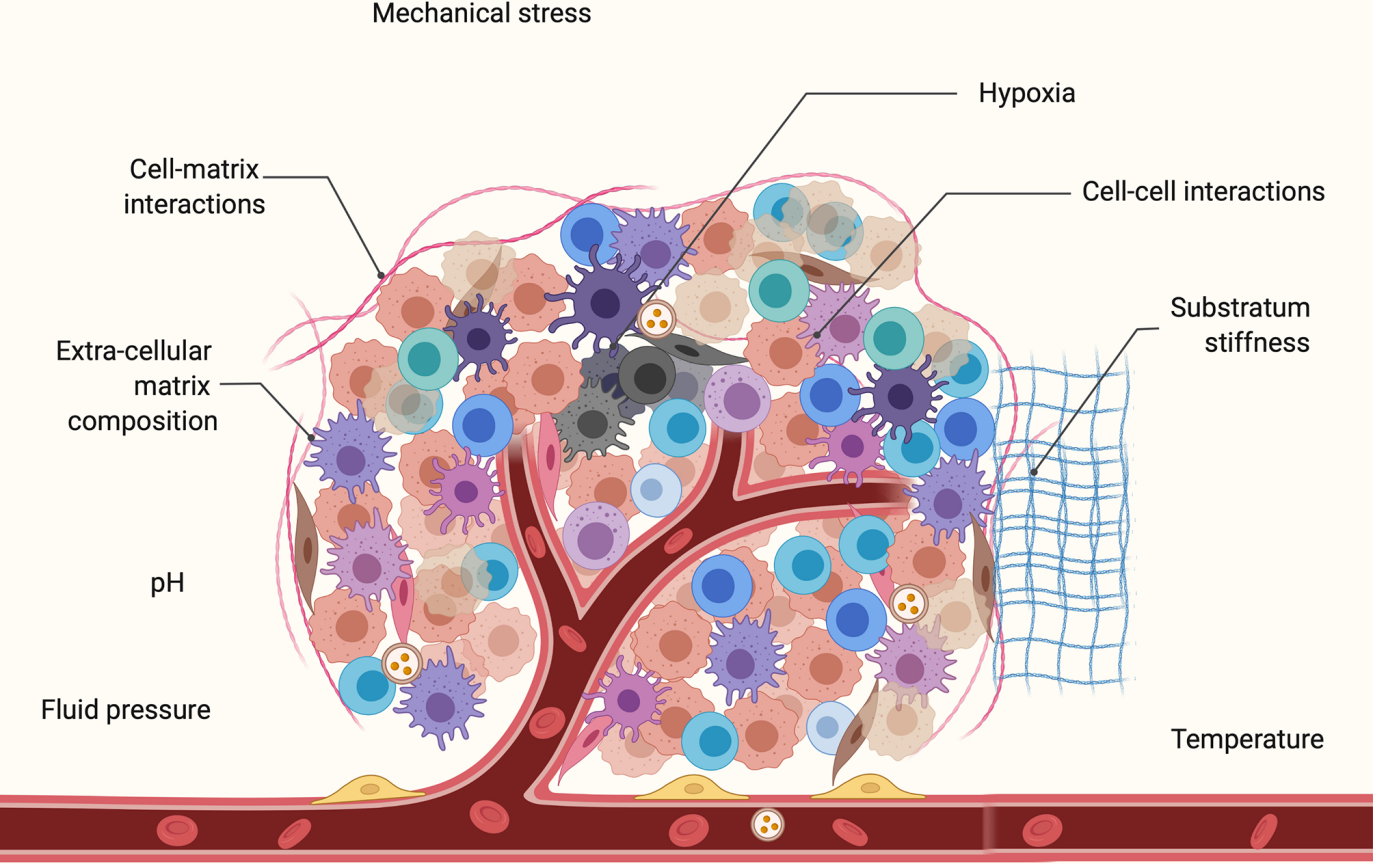
Key components of the tumor microenvironment (TME) which comprises of multiple cell types including cancer cells, immune cells, endothelial cells, and cancer associated fibroblasts. This includes mechanical components such as fluid pressure, substratum stiffness, mechanical stress, cell-cell, and cell-matrix interactions. Also shown are some chemical components of the microenvironment such as pH, temperature, and hypoxic core of the tumor. Cell-cell and cell-TME communication is mediated by a variety of bioactive molecules during carcinogenesis.

The key components of the mechanical microenvironment (**Figure 2**) are solid and fluid pressure, substratum stiffness (120–128) tissue geometry, and mechanical stress (129–131). These components of the physical microenvironment are primarily dependent on mechanosensitive calcium channels Ca_V_3.3 (132, 133). Cells have the ability to sense the surrounding substratum by applying force through actomyosin motors in stress fibers linked to focal adhesions (134). Varying the substratum stiffness has been demonstrated to influence cellular behaviors including differentiation (122), apoptosis (126), proliferation (125), gene expression (135–137), migration (138), cell stiffness (139), and epithelial-mesenchymal transition (EMT) (127). Along with microenvironments of varying rigidity, cells also experience mechanical stress due to the dense packing of neighboring cells. Cell-cell contacts are critical for propagation of bioelectric signals *via* the transport of ions through gap junctions (140–142). The normal breast epithelium cell line MCF10A was demonstrated to respond differently to an EF *in vitro* depending on the confluency of the cell culture (143). The study observed that clustered cells are more sensitive to an EF due to increased cell-cell contacts.

Physical signals from the V_mem_ of the microenvironment also contribute to tumorigenesis (9). Furthermore, pressure activates oncogenic factors such as p38, ERK, and c-Src which are involved in the regulation of cell proliferation, differentiation, and apoptosis (132). Tumors *in vivo* are under higher pressure and are also stiffer than the surrounding tissue which creates a microenvironment that promotes cell proliferation (133). Increased pressure also enhances the invasiveness of tumor cells (121). Additionally, a key communication pathway between cells and their ECM is Integrin signaling pathway which regulates cytosolic Ca^2+^ levels (144). These cytosolic Ca^2+^ concentrations play an important role in cancer-related processes such as EMT (38), metastasis (21), and apoptosis (126, 145). For instance, inducing EMT in human breast cancer cells has been shown to upregulate cytosolic calcium levels (38).

### Cell Migration and Metastasis

**Figure 3:**
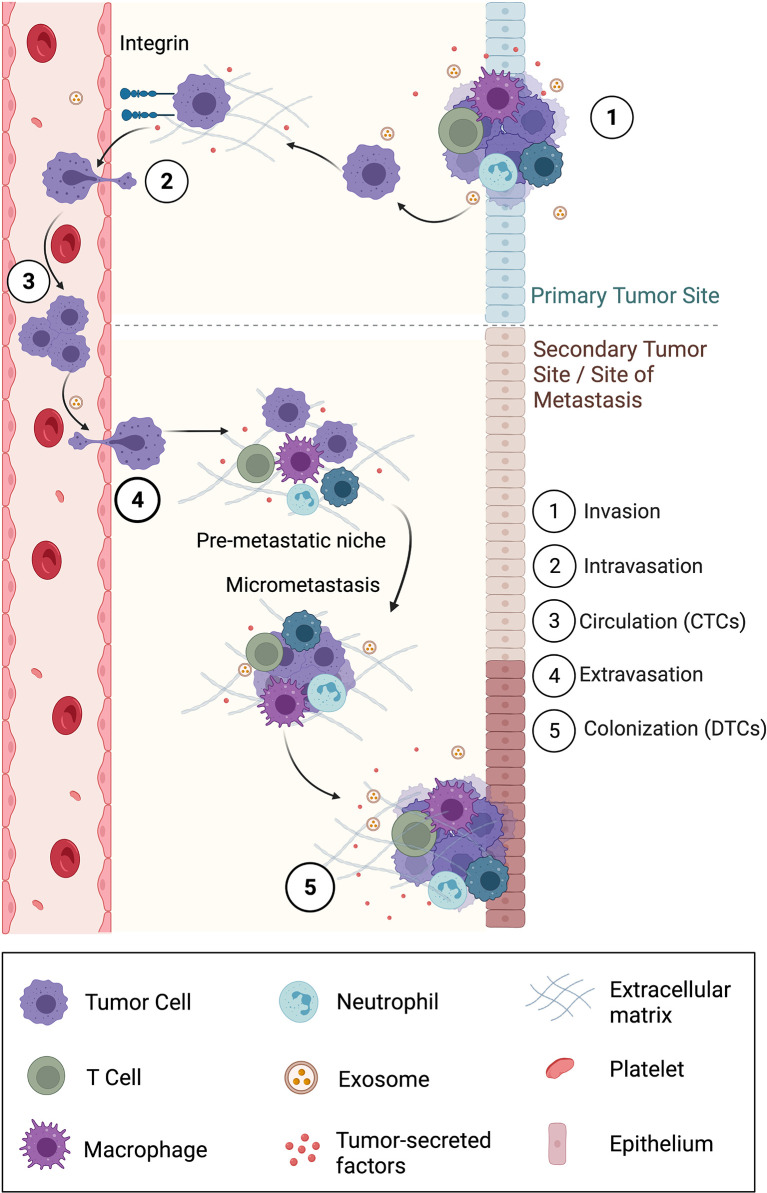
Overview of the five-step metastatic cascade involving local invasion, intravasation into surrounding vasculature, circulation, extravasation, and finally colonization in a secondary location. Also shown is the formation of a pre-metastatic niche that supports the survival of disseminated tumor cells (DTCs) into a successful metastasis. Exosomes, a subpopulation of EVs play a primary role in carrying information from the primary site to the secondary site or site of metastasis, especially to form the pre-metastatic niche.

The dissemination of primary tumor cells to secondary organs is called metastasis. This involves cancer cells breaking away from the primary tumor, traveling through blood or lymphatic systems, and forming secondary or metastatic tumors in other parts of the body. Metastasis is a multi-step process (**Figure 3**) and involves the following events: local invasion to surrounding tissues, intravasation into the vasculature or lymphatics (where they are called circulating tumor cells or CTCs), survival and circulation in the vessels, and extravasation and colonization in a secondary organ (where they are called disseminated tumor cells or DTCs) (146, 147). Bioelectricity mediates many of the normal cell functions which are disrupted in metastasis. Factors such as ion channel expression, V_mem_, and external EFs have been determined to regulate invasion and metastasis. Furthermore, the migration of cancer cells out of the primary tumors into local tissues through various physical barriers is driven by components of the local tumor microenvironment and executed by complex signaling pathways in the cell (10).

Cues within the TME can promote local invasion (148). For instance, an ECM protein fibronectin can attract breast cancer tumor cells to the vasculature *via* haptotaxis (directional migration in response to substrate-bound cues) to promote dissemination. During tumor invasion, constant communication occurs between tumor cells and surrounding stromal cells *via* extracellular vesicles (EVs) (115). Even upon entering a secondary tissue, the transition of a DTC into an overt metastasis is highly dependent on the local microenvironment of this organ (10). Hence, the formation of a supportive premetastatic niche, composed of ECM and resident immune cells is essential to provide nutrients and survival signals that drive DTC survival and outgrowth. Recent work suggests that tumor cells may be able to prime the premetastatic site from a distance before colonization to create a more favorable niche, for example, by secreting exosomes, a subpopulation of EVs (6). Furthermore, even within cancer cells, there is variability in the amount of depolarization. A more depolarized V_mem_ is associated with a higher metastatic potential and forced hyperpolarization of cells can reduce their migration and invasiveness (24, 141, 149, 150).

To study the effect of EFs on cell galvanotaxis, Zhu et al. employed unique probe systems to characterize cancer cell electrical properties and their migratory ability under an EF (151). It was found that tumors established from 4T1, a triple-negative murine breast cancer cell line, produced heterogeneous intratumor potentials causing a flow of endogenous EFs inside and outside of the tumors, which may in turn affect cell migration behavior and ultimately contribute to cancer metastasis. Moreover, tumor electric potentials were found to increase with increase in tumor size, which is an important factor since the primary tumor size has been reported to be linked to the metastatic potential (152, 153). Finally, it was also found that metastatic sublines (m4T1) from lung, heart, axillary lymph node and spleen showed different galvanotaxis thresholds. For instance, parental 4T1 and lung metastatic cell lines were found to respond to EFs as low as 50 mV/mm, while other metastatic sublines showed an anodal migration in a field of 100 mV/mm or higher. Additionally, the migration speeds also varied among different metastatic sublines. Cancer cell monolayers were found to have a higher migration persistence (defined as the ratio of displacement to trajectory length) under EFs than that of isolated cells, suggesting that cancer cells migrated more linearly in a certain direction when responding to EFs collectively.

Interestingly, bioelectric factors override most chemical gradients and other cues in a multi-cue environment during cell migration (8). Lobikin et al. investigated a cell population termed as “instructor” cells which when depolarized, is able to direct the activity of an entirely different set of cells (7). The “instructor” cells induce metastatic phenotype in normal melanocytes by serotonergic signaling, a mechanism which mediates long-range bioelectric signaling. Furthermore, instructor cells also disrupt blood vessel patterning upon depolarization. The melanocytes were then found to acquire three properties commonly associated with metastasis – hyper-proliferation, a highly dendritic morphology, and invasion into tissues such as blood vessels, gut, and neural tube. This data illustrated the power of depolarized V_mem_ as an epigenetic initiator of widespread metastatic behavior in the absence of a centralized tumor.

## Applications

### Current Devices, Materials and Technologies

Molecular-resolution tools have recently been developed for real-time detection and manipulation of bioelectric properties *in vivo* (91, 154). An important component of such investigations is the ability to track spatio-temporal distribution of V_mem_ gradients *in vivo*, over significant periods of time.

#### Detection of Bioelectric Properties

Microelectrodes are a common tool used to measure the electrophysiological characteristics of cells and are extremely powerful for single cell measurements. For instance, Zhu et al. used glass microelectrodes to measure intratumor potentials in subcutaneous tumors established from a triple-negative murine cancer cell line (4T1) (151). However, measurements corresponding to multicellular areas and volumes are constrained by the smaller size of these electrodes. Furthermore, the sample under study needs to be kept perfectly still (154). Fluorescent bioelectricity reporters are a more recent development which has facilitates measurement of electrophysiological properties when it is not feasible to use microelectrodes. These dyes can be used to achieve subcellular resolution, measure many cells simultaneously *in vivo*, and to track bioelectric gradients over long period of time despite cell movements and divisions (154). Chernet and Levin utilized voltage-sensitive fluorescent dyes to non-invasively detect areas of depolarization in oncogene-induced tumor structures in *Xenopus* larvae (24). A few other tools for the characterization of bioelectrical events are highly sensitive ion-selective extracellular electrode probes (105, 155) that reveal ion flux at the cell membrane, reporter proteins (156–159) and techniques that report individual ion species content such as protons (160) and sodium (161). Bioelectronic sensors or biosensors can also be used to sense electric fields, ionic concentrations, and biological markers (162–167). Based on the type of sensor, both intracellular and extracellular recordings of a single cell or a group of cells can be measured. A common transistor biosensor platform used for extracellular recordings is the organic electrochemical transistor (OECT) which is inherently sensitive to ionic species and external electric fields (14). The OECT is typically made of a poly(3,4-ethylenedioxythiophene): polystyrene sulfonate (PEDOT : PSS) mixture and has been implemented for recording of electrochemical gradients in non-excitable cells such as Caco-2 as well as excitable cells (168). Meanwhile, silicon nanowires are suitable for crossing the cell membrane and are commonly used for intracellular readings. These nanowires are synthesized with spatially controlled electrical properties. A nanoscale field effect transistor (NFET) is then created on an individual nanowire by varying the doping levels. NFETs allow localized and tuneable 3D sensing and recording of single cells and even 3D cellular networks. By having a three-dimensional probe presentation, NFETs overcome a major limitation of most traditional nanoelectronic devices which have a more planar design. Tian et al. used three-dimensional NFETs as localized bioprobes for intracellular readings in cardiomyocytes (169). While these methods are excellent tools for measuring cell electrical properties, tools that can manipulate these properties are essential to study the effects of altering cell states.

#### Manipulation of Bioelectric Properties

Bioactuators are a class of devices that can be used to modify cell behavior by delivering directly biophysical signals such as electrophoretic delivery of ions and small molecules targeting specific cell locations (14). Additionally, a variety of nanomaterials have been developed for reading and writing bioelectric cues in tissue. These include biocompatible piezoelectric materials and nanoparticles that alter the resting potential of cells by contact, without the use of transgenes (170–173). Warren and Payne determined that nanoparticles with amine-modified surfaces induced significant depolarization in both, Chinese Hamster Ovary (CHO) cells and HeLa cells (173). Conductive polymers are another class of materials that can stimulate cells or tissue cultured upon them (174–176) by applying an electrical signal. Conductive polymers used in tissue engineering include conductive nanofibers, conductive hydrogels, conductive composite films, and conductive composite scaffolds fabricated using methods such as electrospinning, coating, or deposition by *in situ* polymerization (177). For instance, Jayaram et al. used PEDOT : PSS conducting polymer microwires to depolarize cells and achieve a more positive membrane potential in *E. coli* cells (170). Thourson and Payne also demonstrated the use of PEDOT : PSS microwires to control action potentials of cardiomyocytes (178). Conductive polymer microwires thus provide a minimally invasive platform to control electrical properties of cells with high spatial precision. Detailed reviews on conductive polymers have been previously published (177, 179).

**Figure 4:**
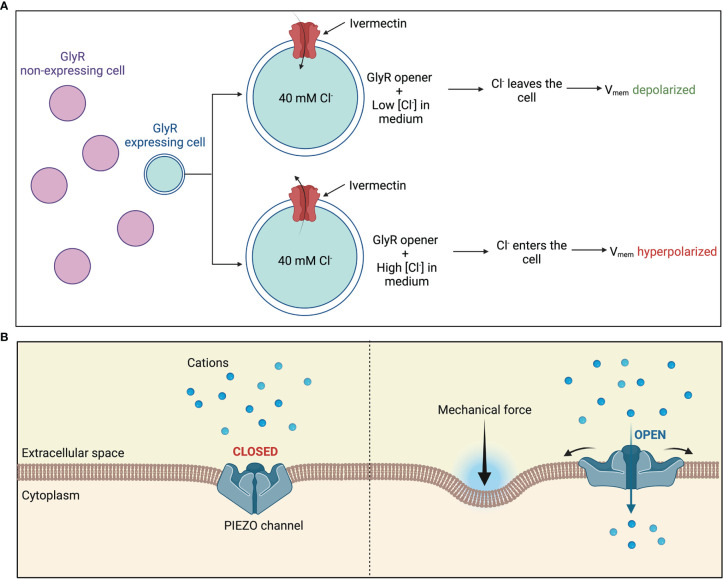
Manipulating bioelectric properties of cells **(A)** Manipulation of endogenous chloride channels as a means of manipulating V_mem_ of a select group of cells. Treatment with ivermectin causes chloride channels in GlyR-expressing cells to open. External chloride levels are then manipulated to regulate movement of chloride flux into or out of the cytoplasm **(B)** Piezo1 and Piezo2 are mechanically activated cation channels. Application of a mechanical force causes the central pore to open, allowing an influx of positive charge into the cell.

As mentioned previously, treatment with ivermectin is another way to control the transmembrane potential of a select group of cells by manipulating of endogenous chloride channels (**Figure 4A**). Ivermectin targets GlyR-expressing cells and hence opens their chloride channels. Chloride ions can then be made to enter or exit the GlyR-expressing cells by manipulating the external chloride levels, thus controlling their transmembrane potential (7). For instance, a low level of chloride in the external medium would cause chloride ions to exit the cell and into the medium, hence depolarizing the cell. Lobikin et al. employed this method in frog models to regulate the membrane potential of a specific group of cells expressing GlyCl channels to desired levels and study the consequences on metastasis and tumorigenesis *in vivo.*

Another potential way to manipulate the bioelectric properties of cells is by controlling the mechanosensitive Ca^2+^-permeable Piezo channels which have emerged as major transducers of mechanical stress into Ca^2+^ dependent signals. These mechanosensitive Piezo channels expressed on the plasma membrane are gated by various mechanical stimuli such as stiffness, compression, tension forces, and shear stress. Channel activation then allows a Ca^2+^ influx into the cytoplasm which then mediates the cell polarity (**Figure 4B**). Piezo1 may also be pharmacologically activated by agonists such as Jedi1, Jedi2 and Yoda1 or inhibited by channel pore blockers, competitive antagonists, and peptides such as Ruthenium Red, GsMTx-4, Dooku1 and Aß peptides which distort the membrane mechanical properties (87). Han et al. demonstrated that activation of Piezo1 *via* mechanical stimuli in 1 μm using a heat-polished glass probe controlled by a piezo electric device or *via* agonist Yoda1 mediated Ca^2+^ influx in pancreatic cancer cells, resulting into a more depolarized state (88).

### Extracellular Vesicles and Electricity

Extracellular vesicles (EVs) facilitate inter-cellular communication *via* delivery of proteins and nucleic acids, including microRNA (miRNA) and mRNA (180). EVs-mediated communication is vital during the establishment of planar cell polarity and the developmental patterning of tissues (181). EVs are particularly enriched in the tumor microenvironment (182, 183) and as mentioned in the previous sections of this paper, they play a special role in cancer development and progression. In a recent study, Fukuta et al. demonstrated that external stimuli such as low levels of electric field treatment that activate intracellular signaling would likely increase exosome secretion from the cells. It was seen that an electric field of 0.34 mA/cm^2^ increases the secretion of these EVs from cultured cells of murine melanoma B16F1 and murine fibroblast 3T3 Swiss Albino without compromising their quality (180). These results together indicate that the bioelectric dysregulation or depolarization of cells that occurs during cancer may be responsible for the upregulation of EVs in the cancer tumor microenvironment. At the same time, the increase in production of EVs plays a role in disrupting the bioelectric homeostasis, forming a feedback loop. The change in cell state and EV production along with the interdependence of the two are major mechanisms responsible for the bioelectric dysregulation of cancer.

## Conclusions

Bioelectric signaling is a growing field of study that takes us a step closer to understanding cancer as a disease, all the way from initiation to metastasis. A lot is known about cancer and its biology as per the somatic mutation theory. On the other hand, the role of electric fields in cancer processes, while strongly established over the last few decades, needs further investigation. Understanding the bioelectric mechanisms underlying cancer is especially important since it will allow us to develop new biomedical and bioengineering tools and techniques as per the tissue organization field theory. These new engineering tools, along with the existing biological knowledge will enhance our understanding of cancer and enable the development of novel treatments for patients.

Another exciting area of study is the interplay between the bioelectric dysregulation and enhancement of extracellular vesicles (EVs) within the context of the cancer microenvironment. It has been well established that EVs play a significant role in facilitating the signaling pathways involved in all processes of carcinogenesis. This paper provides a detailed review of the current knowledge about bioelectric dysregulation that underlies different processes of cancer. However, little is known about the interdependence of these two mechanisms. Furthermore, EVs, especially exosomes, have been proven to have a role in therapeutic strategies for cancer. Understanding this crosstalk will not only enhance our knowledge of cancer, but also help develop efficient exosome-based cancer immunotherapies and drug delivery vehicles.

## Author Contributions

MS wrote the main manuscript text and prepared all figures. LE gave suggestions and ideas on the literature search and manuscript writing. All authors contributed to the article and approved the submitted version.

## Funding

This work has been funded by the National Science Foundation NSF CAREER ECCS (2046037).

## Conflict of Interest

The authors declare that the research was conducted in the absence of any commercial or financial relationships that could be construed as a potential conflict of interest.

## Publisher’s Note

All claims expressed in this article are solely those of the authors and do not necessarily represent those of their affiliated organizations, or those of the publisher, the editors and the reviewers. Any product that may be evaluated in this article, or claim that may be made by its manufacturer, is not guaranteed or endorsed by the publisher.

## Abbreviations

EV, extracellular vesicle; TME, tumor microenvironment; V_mem_, membrane potential; EF, electric field; SMT, somatic mutation theory; TOFT, tissue organization field theory; HMEC, human mammary epithelial cell; MCF7, estrogen-receptor-positive breast cancer cell line; MDA-MB-231, estrogen-receptor-negative breast cancer cell line; ECM, extracellular matrix; EMT, epithelial mesenchymal transition; CAF, cancer associated fibroblast; GlyCl, glycine-gated chloride channel; VGCC, voltage gated calcium channel; VGSC, voltage gated sodium channel; EAG, ether-à-go-go K^+^ channel; TRP, transient receptor potential; MAPK, mitogen-activated protein kinase; JNK, c-Jun N-terminal kinase; ERK, extracellular signal-regulated kinase; Pl3K/Akt, phosphatidylinositol 3-kinase/protein kinase B; MEK, mitogen-activated ERK kinase; EGF, epidermal growth factor; EGFR, epidermal growth factor receptor; GBM, glioblastoma multiforme; GSK-3ß, glycogen synthase kinase-3 ß; ITLS, induced tumor like structure; MCF10A, normal breast epithelium cell line; CTC, circulating tumor cell; DTC, disseminated tumor cell; mRNA, messenger RNA; miRNA, micro RNA; B16F1, murine melanoma cell line; 3T3, murine fibroblast cell line; OECT, organic electrochemical transistor; CHO, Chinese hamster ovary; PEDOT : PSS, poly(3,4-ethylenedioxythiophene): polystyrene sulfonate.
